# Complications and Diagnosis of Branchial Cleft Cysts: A Case Report

**DOI:** 10.7759/cureus.32667

**Published:** 2022-12-18

**Authors:** Sean M McCormack, Maria J Nicewicz

**Affiliations:** 1 Pediatrics, Saint James School of Medicine, Park Ridge, USA; 2 Pediatrics, Resurrection Medical Center, Chicago, USA

**Keywords:** pediatric deep neck space infections, congenital disease, radiological evaluation, radiological investigations, radiological findings, incidental radiological finding, acute pharyngitis, deep neck space abscess, branchial cleft cyst, branchial cleft anomaly

## Abstract

Branchial cleft cysts (BCCs) are congenital anomalies that can be found in children and young adults. The exact incidence of these anomalies is unknown as the diagnosis may be missed. Branchial cleft cysts can present in a variety of locations depending on the cleft they are derived from. Regardless of location, branchial cleft cysts are rather benign. However, a variety of complications can arise due to infection, and infections are often recurrent. Diagnosis may occur incidentally on imaging studies as such studies are often performed to rule out a variety of complications from infections alone. Treatment includes first treating any infection and any such complications that exist, followed by surgical excision. Surgical excision is performed to prevent the recurrence of infection. A case of a 14-year-old female with a painful swollen throat, trismus, and difficulty swallowing is reported.

## Introduction

Before antibiotic use became widespread, most deep neck space infections were caused by the spread of tonsillar and pharyngeal infections, e.g., tonsillitis and pharyngitis. To date, tonsillitis still is the cause of most deep neck infections in pediatric populations [1,2]. Branchial cleft cysts (BCCs) are a unique risk factor for deep neck infection even though they are rather benign in nature. However, when treating deep neck infections, it is important to treat the underlying cause of the infection. In this case, we will look at a presenting risk factor for severe deep neck infection in this pediatric patient [3].

A case of a 14-year-old female with painful neck swelling, fever, and difficulty swallowing is reported. The etiology was ultimately determined to be secondary due to pharyngitis complicated by a BCC infection. BCCs are the result of the congenital failure of involution. These congenital malformations are prone to infection if left untreated. While these cysts are easily treatable, they may not be obvious, and complications can occur during excision. Surgical excision is the definitive treatment to prevent recurrent infections and additional complications such as deep neck abscess [4-6].

## Case presentation

A 14-year-old female presented to the clinic with her mother due to complaints of a painful swollen neck, throat pain, fever (maximum temperature of 102.3°F), and increasing difficulty swallowing for four days. The patient described the pain as severe and causing a challenge to both breathing and swallowing. She denied cough, vomiting, chest pain, shortness of breath, and diarrhea. Upon arrival, the patient was febrile with a low-grade fever of 100°F, nonlabored respirations, a blood pressure of 112/70 mmHg, an oxygen saturation (SPO_2_) of 98% on room air, and no acute distress. The patient reported difficulty tolerating even soft foods and liquids. As per the patient, this was the first time she had an illness with these symptoms. Her symptoms had a rapid onset and had not been getting better over the past four days; rather, she had been deteriorating. A physical examination revealed a pale, well-nourished adolescent in great discomfort.

On physical examination, the patient’s general appearance was fatigued, uncomfortable, and with pale skin. The patient’s neck was tender with large amounts of soft tissue swelling. Swelling was present on the anterior part of the neck in the submandibular area. Pain was most severe in this region of the neck; however, the patient noted more diffuse neck pain. Swelling and pain interfered with lymph node palpation. Pain and trismus limited the oral examination and the patient’s willingness to participate in the examination. The patient was unable to open her mouth wide enough for proper examination but was able to support her airway.

Due to severe neck swelling with difficulty swallowing, both the patient and parent were advised to go to the emergency room. The patient and the parent were educated about complications; however, both refused to go to the emergency room as they were medically advised. The patient preferred to be treated in the outpatient setting than be admitted to the hospital. Rapid tests for COVID-19 and influenza were negative. Rapid testing for group A strep was forgone as the patient was unable to open her mouth. Laboratory results were drawn including complete blood count (CBC), comprehensive metabolic panel (CMP), C-reactive protein (CRP), erythrocyte sedimentation rate (ESR), Epstein-Barr virus (EBV) heterophile antibody, EBV IgG, and EBV IgM, and the viral panel was collected by PCR. The patient refused throat culture. The patient was sent for a stat ultrasound and stat X-ray of the neck to evaluate for complications of pharyngitis including deep neck abscess. The findings were not immediately available and will be discussed later in this case report. The patient subsequently received empiric antibiotic treatment with intramuscular (IM) ceftriaxone 500 mg due to the inability to tolerate capsules or liquids orally. The patient and the mother were given directions to return to the clinic on the next day for a follow-up and to present to the emergency room if symptoms worsen or if the patient experiences any difficulty breathing.

The patient returned to the clinic the following day with extensively improved symptoms of soft tissue neck swelling, pain, fever, and trismus and an increased ability to swallow. The patient was more willing to participate in the physical examination due to improved symptoms. Examination findings showed a slightly symmetrical face with swelling still present more so on the left. Submandibular lymph nodes and left lateral cervical lymph nodes were enlarged and tender to palpation. Soft tissue swelling was improved, with the swelling lateralized to the left. Pain was still present in the submandibular area especially on the left. The throat showed an erythematous oropharynx, with some erosion of the pharyngeal arch. Laboratory results were negative for EBV heterophile antibodies, EBV IgG antibodies, EBV IgM antibodies, and other common viral infections via PCR. Bloodwork showed normal white cell count (5.2 g/L), normal hemoglobin (13.2 g/dL) and hematocrit (39.6%), elevated sedimentation rate (82 mm/hour), and elevated C-reactive protein (93.3 mg/L). All other results were within normal limits. Laboratory results can be further visualized in Table 1. The results of imaging studies were still pending. Due to immensely improved symptoms with intramuscular antibiotics, empiric antibiotic treatment was continued. The patient was prescribed cefdinir 300 mg oral capsules for 10 days due to amoxicillin allergy. The patient was able to tolerate capsules at this time. The patient was directed to return to the clinic in two days.

**Table 1:** Laboratory values.

Values	Day 1	Follow-up
Leukocyte count (/mm^3^)	10.6	5.2
Hemoglobin, blood (g/dL)	14	13.2
Hematocrit (%)	41.2	39.6
Erythrocyte sedimentation rate (mm/hour)	82	72
C-reactive protein (mg/L)	93.3	20.8

On two-day follow-up, the patient showed continued improvement of symptoms, with pain almost exclusively on palpation of the submandibular area. Lymph nodes were noted to still be enlarged. Sedimentation rate and C-reactive protein had improved to 72 mm/hour and 20.8 mg/L, respectively (Table 1). 

Several radiological studies were ordered stat on the first day to rule out complications of pharyngitis. Complications including peritonsillar abscess, retropharyngeal abscess, and parapharyngeal abscess are important to consider given the examination findings of neck swelling and trismus. Ruling out these differentials requires a thorough workup. The results of these studies were useful in ruling out complications of pharyngitis and identifying underlying pathology.

**Figure 1:**
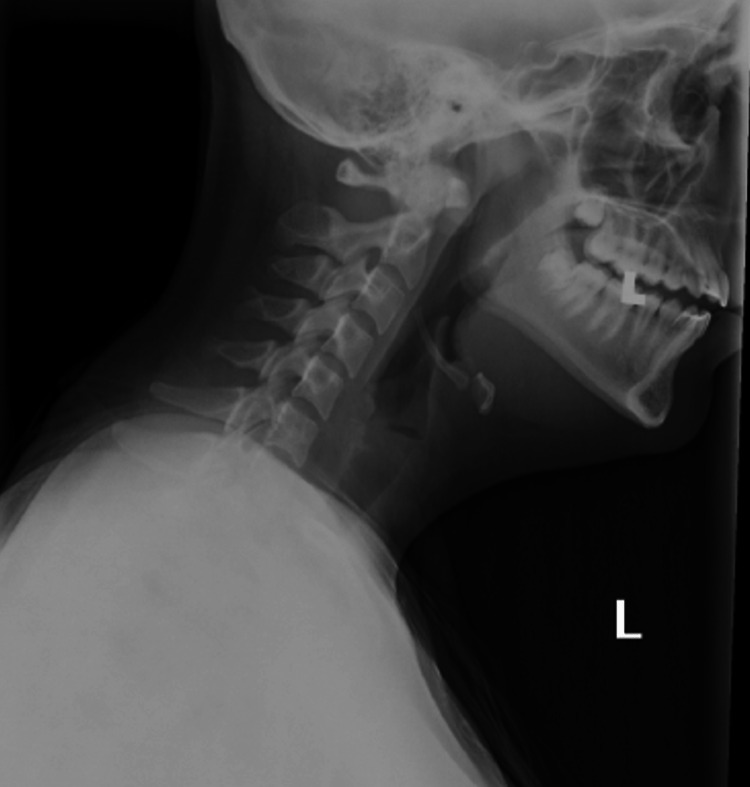
Cervical spine radiograph (lateral view).

**Figure 2:**
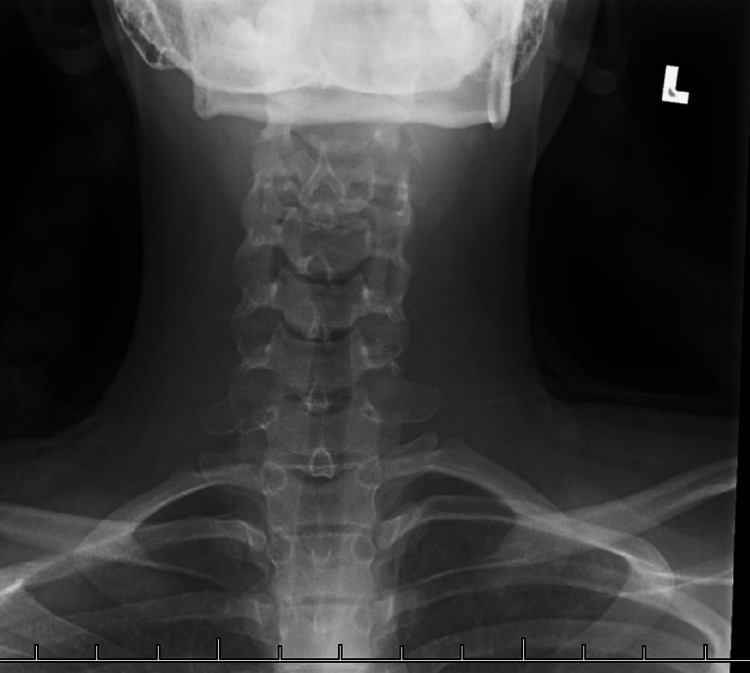
Cervical spine radiograph (AP view). AP: anteroposterior

Neck X-ray showed no significant findings suggestive of an underlying pathologic process. X-rays of the neck are useful for ruling out retropharyngeal abscess. There was no evidence of prevertebral swelling on imagining, excluding retropharyngeal abscess from the differential. The epiglottis was also seen to be normal without any significant subglottic narrowing (Figures 1-2).

**Figure 3:**
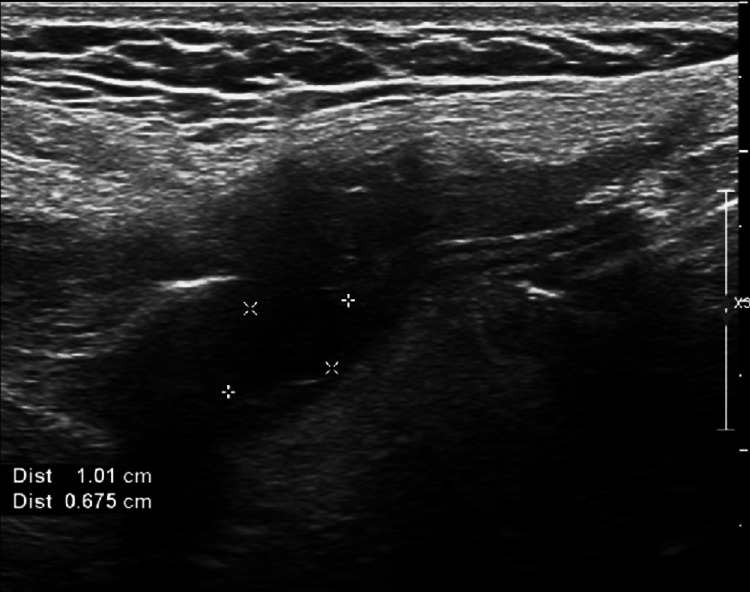
Ultrasonography of neck mass (longitudinal view).

**Figure 4:**
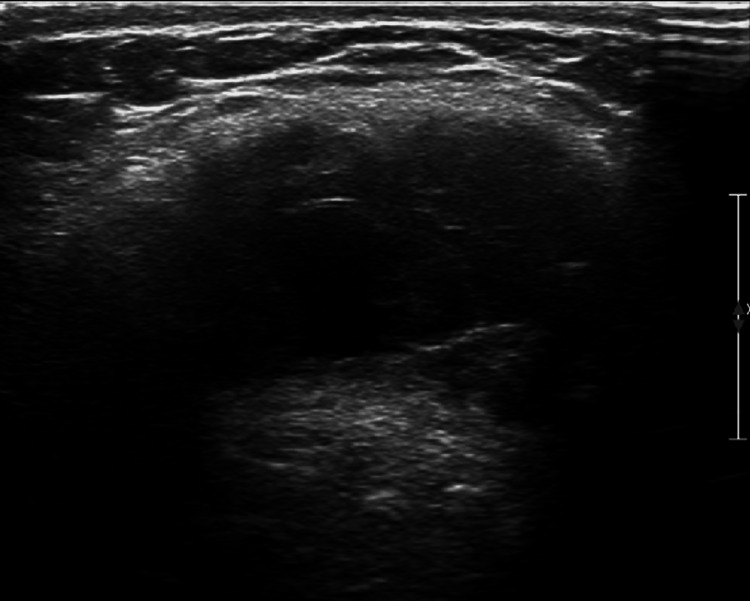
Ultrasonography of neck mass (transverse view).

**Figure 5:**
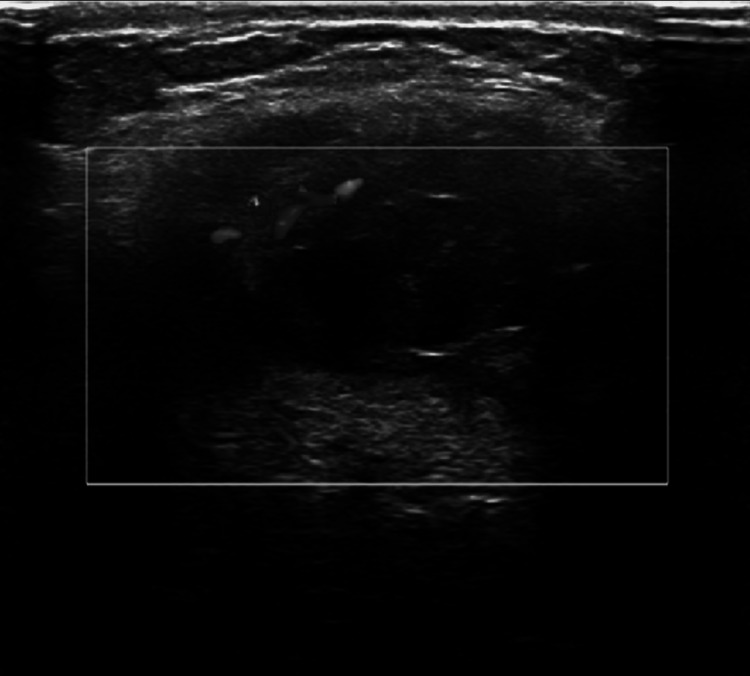
Ultrasonography of neck mass (transverse Doppler view).

Neck ultrasound had significant findings. A discrete well-circumcised hypoechoic lesion with an echogenic rim was seen in the soft tissues of the anterior part of the neck deep to the subcutaneous tissues. There was no vascular flow within this lesion on Doppler imaging. The lesion measured 1.0 cm in diameter. This was suggestive of a small fluid collection or a congenital anomaly such as a BCC. No adenopathy was seen on sonography (Figures 3-5).

Due to the findings on ultrasonography, the patient was referred to computed tomography (CT) with intravenous (IV) contrast to further investigate the ultrasound findings and to an otolaryngology specialist as well. Again, the mother was educated about the possibility of recurrence of infections. However, due to an excellent recovery from the patient, no history of recurrent infections, and the discontinuation of clinical symptoms, the mother decided to defer further investigative imaging studies at this time.

## Discussion

Origin

BCCs are remnants of the embryological branchial cleft or cervical sinus, which normally regresses before birth. When a branchial cleft does not properly regress, a BCC forms. Second BCCs are the most common type of branchial cleft anomaly; however, there are cases where the origin comes from the first, third, or fourth cleft. Branchial sinuses and fistulae are other congenital anomalies due to brachial malformations.

First BCCs are located near the external auditory canal or the angle of the mandible. First BCCs are the second most common type of BCC. Second BCCs are located inferior to the angle of the mandible and near the anterior border of the sternocleidomastoid muscle. However, cysts derived from the second branchial cleft can be found anywhere along the course of a second branchial fistula, which runs between the internal carotid and external carotid toward the palatine tonsil. The location of this cyst makes it an important differential when evaluating parapharyngeal masses. Third BCCs can be located anywhere along a fistula similarly to second BCCs. However, the tract is found posterior to the carotid arteries and deep to the sternocleidomastoid muscle. Third BCCs are rare. Fourth BCCs can be located anywhere along a fistula similar to the cysts derived from other clefts. This tract is located in the lateral neck near the recurrent laryngeal nerve. Fourth BCCs are also rare [4,7].

Occasionally, a branchial pouch and a branchial cleft both fail to regress, and a complete fistula forms between the pharynx and the skin. This is also rare. A fistula to the skin more often forms due to recurrent infections of the cyst; this complicates treatment, and damage to nearby structures becomes more likely [4,7].

BCCs usually present in late childhood or early adulthood when a previously unrecognized cyst becomes infected. They are characterized as lateral neck masses; this distinguishes them from other conditions that present in the midline such as thyroglossal duct cyst. BCCs are also characterized as painless, mobile swellings. However, recurrent infection is common, and these masses can become painful when infected. It is important to differentiate BCCs from other deep neck infections such as parapharyngeal abscess, retropharyngeal abscess, and peritonsillar abscess as the treatment for BCCs requires a different treatment [8,9].

Complications

Branchial anomalies are the result of the failure of involution of biological remnants of our evolutionary predecessors. These lateral neck masses are risk factors for infection following illness such as, but not limited to, tonsillitis, pharyngitis, dental infections, oral surgical procedures, salivary gland infection, trauma, instrumentation, foreign body aspiration, cervical lymphadenitis, or just idiopathic. Recurrent infection can cause further damage to nearby anatomical structures and create a fistula to the skin. Diagnosis includes both clinical findings and radiological imaging to rule out other sources of infection. Imaging is also useful in planning for surgical intervention [5,6].

Differential

When a patient, either pediatric or adult, presents with signs of infection and neck swelling, deep neck infections should always be included in the differential diagnosis. Deep neck infections including peritonsillar abscess, retropharyngeal abscess, and parapharyngeal abscess make up 3,400 hospitalizations every year in the United States [10]. The symptoms of deep neck infections include dental pain, dysphagia, stridor, drooling, inability to swallow, dysphonia, trismus, torticollis, pain on neck movements, dysphonia, hoarseness, and respiratory distress. Physical examinations may show findings such as facial/neck asymmetry, redness, swelling, induration, and regional lymphadenitis [2,11]. Deep neck infections can be difficult to diagnose based on clinical findings alone, but infection can be demonstrated easily on imaging, including CT. Physicians should not hesitate to order imaging in the absence of life-threatening symptoms. For patients with the most severe symptoms, the operating room, where an artificial airway can be placed if needed, is the proper setting for evaluation, especially when life-threatening symptoms such as airway compromise are of concern [8].

Plain radiography of the lateral neck can be used to assess for retropharyngeal abscess; the widening of the prevertebral space in the cervical area is suggestive of retropharyngeal abscess. This can be well visualized on lateral view cervical radiographs [10,12].

Ultrasound can be useful to rule out phlegmon and is an accurate means of evaluating patients for peritonsillar abscess. Ultrasound has been shown to be an effective imaging modality to rule out peritonsillar abscess in pediatric patients [13]. However, ultrasound may not provide enough information on its own in some cases. This is especially true for deep infections [10]. Ultrasound is also a first-line imaging method of choice for defining benign lesions that are cystic in nature such as what is presented in this case. The lack of Doppler flow is useful in distinguishing a BCC from hemangioma, lymphoma, or other rare conditions such as branchiogenic carcinoma. Ultrasound is a routinely used imaging modality and may indecently find a congenital neck mass. Sonography can confirm a diagnosis of a BCC and differentiate it from other pathologic findings; however, it may be unable to delineate the entire lesion [13-16].

CT with IV contrast is the preferred imaging modality for identifying deep space neck infections, as well as congenital neck masses. There is little utility in obtaining a non-contrast CT scan as phlegmon and abscess are not easily differentiable. Magnetic resonance imaging (MRI) is also useful in diagnosing deep neck infections. MRI has been shown to confirm neck infections in more than 95% of patients suspected to have deep neck infections. Significant cost differences, imaging times, widespread availability, and the ease of access between CT and MRI influence clinical decision-making when ordering tests. Both CT and MRI tests are effective imaging modalities; however, CT is used more frequently to aid diagnosis or to rule out deep neck infections due to the aforementioned logistical differences. When coming to a definitive diagnosis, the location, clinical picture, and radiological correlation are all important factors to consider [4,10,17].

Unfortunately, follow-up imaging in this case to determine a definitive diagnosis was not possible due to noncompliance. However, due to the characteristics seen on ultrasound imaging, the clinical picture, and the location of the mass, we arrived at a plausible diagnosis of a BCC.

Treatments

The management of BCCs begins with controlling any underlying infection, if present. Antibiotic therapy is used for uncomplicated infections. An abscess, particularly a large abscess, may require incision and drainage. Incision and drainage is then followed by antimicrobial therapy. Once the infection has resolved, the mass is usually excised to prevent future problems [4,5]. Without complete excision of the entire tract or sinus, recurrent infections are common, and repeated incision and drainage may be needed. This is due to the recurrence of infection within the congenital anomaly. Due to this, complete excision is generally preferred. The procedure includes a series of horizontal incisions to fully dissect out the convoluted path of the BCC. The most common complication of surgery is scarring or complications due to the damage to surrounding structures. Scarring is relatively significant in many cases. Other rare complications have been reported including pressure symptoms, pain, and superimposed infection [18,19].

Sclerotherapy and endoscopic excision are other treatment options depending on the size and location of the lesion. Alternative treatment is limited due to incomplete visualization of the lesion. These alternative treatment options are of use if open neck surgery needs to be delayed; it is recommended that surgery be delayed until a patient is at least three months of age. Acute infection and abscess are other contraindications to open neck surgery, and alternative treatments may be useful on a case by case basis. If an abscess is present, surgical incision and drainage is indicated, along with antimicrobial therapy prior to excision [18,19].

## Conclusions

BCCs are indolent masses and are most often found before adulthood. BCCs are most commonly derived from the second branchial cleft. BCCs are a unique risk factor for developing deep neck infection. Additionally, infected BCCs present in a similar fashion to deep neck infections. Due to the similarities between the two, BCCs are usually found incidentally during the workup of deep neck infections on imaging studies or due to repeated infections. A case where a cystic structure suggestive of a BCC is found incidentally on ultrasound following a severe case of pharyngitis in an adolescent is presented here. Treatment is rather straightforward once the diagnosis is confirmed. Treatment involves ruling out any neck abscess and treating any underlying infection, followed by complete excision of the cyst.
